# *SVALKA*: A Long Noncoding Cis-Natural Antisense RNA That Plays a Role in the Regulation of the Cold Response of *Arabidopsis thaliana*

**DOI:** 10.3390/ncrna10060059

**Published:** 2024-11-28

**Authors:** Nicholas M. Kiger, Susan J. Schroeder

**Affiliations:** 1 School of Biological Sciences, University of Oklahoma, Norman, OK 73019, USA; nicholas.m.kiger-1@ou.edu; 2 Department of Chemistry and Biochemistry, University of Oklahoma, Norman, OK 73019, USA

**Keywords:** environmental stress response, gene regulation, long noncoding RNA

## Abstract

RNA plays important roles in the regulation of gene expression in response to environmental stimuli. *SVALKA*, a long noncoding cis-natural antisense RNA, is a key component of regulating the response to cold temperature in *Arabidopsis thaliana*. There are three mechanisms through which *SVALKA* fine tunes the transcriptional response to cold temperatures. *SVALKA* regulates the expression of the *CBF1* (C-Repeat Dehydration Binding Factor 1) transcription factor through a collisional transcription mechanism and a dsRNA and DICER mediated mechanism. *SVALKA* also interacts with Polycomb Repressor Complex 2 to regulate the histone methylation of *CBF3*. Both *CBF1* and *CBF3* are key components of the *COLD REGULATED* (*COR)* regulon that direct the plant’s response to cold temperature over time, as well as plant drought adaptation, pathogen responses, and growth regulation. The different isoforms of *SVALKA* and its potential to form dynamic RNA conformations are important features in regulating a complex gene network in concert with several other noncoding RNA. This review will summarize the three mechanisms through which *SVALKA* participates in gene regulation, describe the ways that dynamic RNA structures support the function of regulatory noncoding RNA, and explore the potential for improving agricultural genetic engineering with a better understanding of the roles of noncoding RNA.

## 1. Introduction

The proliferation of next-generation sequencing techniques has revealed many different functional classes of RNA [1,2]. The most recent *Arabidopsis thaliana* transcriptome includes 14,880 non-protein coding genes, which is 8.8% of all annotated transcripts [2]. One type of regulatory noncoding RNA is long noncoding RNA. Long noncoding RNAs (lncRNAs) have two key features [3,4,5,6]. lncRNAs have a length greater than 500 nucleotides and are unlikely to be translated, as defined by the lncRNA community consensus statement [6]. lncRNAs are transcribed by RNA polymerase II (RNAPII) or RNA polymerase IV. lncRNAs are often spliced, capped, and polyadenylated. lncRNAs can be further subdivided into categories based on their major features, including genomic location and context, effect on DNA sequences and transcription, and mechanism of functioning [5,7,8]. Examples include intergenic lncRNAs (lincRNAs), natural antisense lncRNAs (NAT-lncRNAs), and intronic lncRNAs (lincRNAs).

This review will focus on *SVALKA*, its mechanisms of action, potential for functional RNA structure, and potential for improving crop stress tolerance. Many outstanding reviews aptly summarize the current state of plant lncRNA research in response to environmental stress, methods for lncRNA discovery, and the gene regulation pathways for cold response [3,6,9,10,11,12,13,14,15,16]. Many reviews of the cold response in plants focus on protein transcription factors. This review takes an RNA-centric view and focuses on *SVALKA*. First, we describe the present knowledge of *SVALKA* mechanisms of action [17,18,19]. Second, we discuss possible RNA structure–function relationships regarding *SVALKA*. Dynamic RNA structures can direct RNA function and protein binding interactions [20]. We summarize how structure directs RNA function in other lncRNAs that mediate cold response, such as *COOLAIR*, *COLDAIR*, and *COLDWRAP*. Next, we discuss the potential applications of *SVALKA* to improve agricultural bioengineering. We highlight the plant cold response framework within which *SVALKA* functions and discuss past research attempts to genetically engineer more cold-resistant crops prior to the discovery of *SVALKA*. Finally, we provide perspectives on how *SVALKA* might be leveraged to aid in the creation of cold-tolerant plants in the future. Thus, the aim of this review is to connect molecular mechanisms of gene regulation, RNA structure, and agricultural applications in bioengineering for *SVALKA* as a specific example of a lncRNA that contributes to environmental stress response in plants.

**Figure 1:**
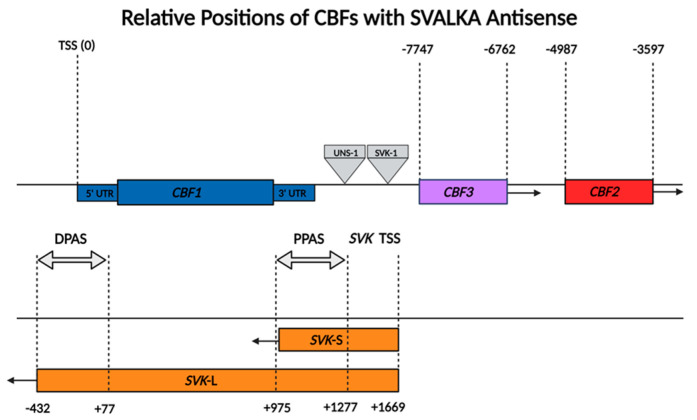
Illustration of the relative locations of the *CBF* cluster and *SVALKA* genes to each other. The two *SVALKA* isoforms, *SVK*-L and *SVK*-S, are shown. The transcription start sites (TSSs) for *CBF1* and *SVALKA* are given, and the numbers given are the nucleotides up/downstream of the TSS for *CBF1*, showing the location of the distal polyadenylation site (DPAS) and proximal polyadenylation site (PPAS) corresponding to *SVK*-L and *SVK*-S, respectively. The relative locations of the *svk*-1 and *uns-1* (uncoupling *SVALKA* 1) T-DNA inserts are indicated. Figure drawn approximately to scale. Created with BioRender.com.

*SVALKA*, Swedish for “cool”, is a cis-natural (*cis*-NAT) antisense transcript lncRNA first identified in *Arabidopsis thaliana*. *Cis*-NAT lncRNAs overlap and are complementary to the gene they regulate but are transcribed from the opposite DNA strand. *SVALKA* is transcribed proximally and antisense to the genes it regulates, *CBF1* and *CBF3* (Figure 1). Like all lncRNAs, *SVALKA* does not show appreciable levels of translation, and exists in two main isoforms that are both larger than 500 nucleotides [17]. *SVALKA* exists as a long isoform (*SVK*-L) of 2,102 nucleotides and a short isoform (*SVK*-S) of 696 nucleotides [17]. *SVALKA* is transcribed by Pol II. Transcriptional read through by Pol II from the *SVALKA* transcription start site generates a cryptic antisense RNA (*asCBF1*) that overlaps the protein-coding regions of *CBF1* [17].

## 2. *SVALKA*, a Long Noncoding RNA, Regulates Gene Expression in Response to Cold Using Three Distinct Mechanisms

*SVALKA* governs precise adjustments to *CBF1* expression at a range of temperatures. Figure 1 shows the relative genomic positions of SVALKA to the two genes that it regulates, *CBF1* and *CBF3*. Two main isoforms make up the majority of *SVALKA* transcripts. *SVK*-L and *SVK*-S predominate at 4 and 22 °C, respectively. Both fine tune *CBF1* expression but each isoform uses a different mechanism. Both isoforms of *SVALKA* are polyadenylated, with a different polyadenylation site associated with each isoform. At 4 °C, the proximal poly(A) site dominates for *SVK*-S transcription. At 22 °C, the distal poly(A) site dominates for *SVK*-L transcription.

**Figure 2:**
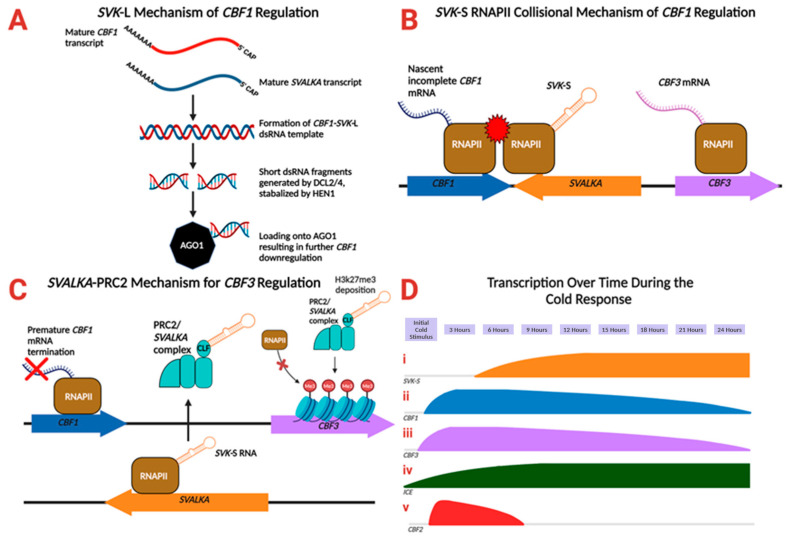
The three different known mechanisms of *SVALKA* regulation. (**A**) Mechanism by which *SVK*-L adopts a dsRNA (double-stranded RNA) conformation with *CBF1* mRNA and regulates *CBF1* expression at 22 degrees Celsius. (**B**) *SVK*-S RNAPII collision-based mechanism of regulating *CBF1* in response to cold stress (4–8 h after freezing exposure). Sense/antisense collision of *CBF1/SVALKA* RNAPII occurs, resulting in premature transcript termination. Note that although they are shown on the same strand here, *SVALKA* is antisense to *CBF1*. Prior to Polycomb Repressive Complex 2 (PRC2) recruiting, *CBF3* is transcribed regularly. *SVALKA* lies between *CBF1* and *CBF3*, but antisense to them. (**C**) *SVALKA*-PRC2 mechanism for methylation of CBF3 (24 h after freezing exposure). *SVALKA* RNA recruits PRC2 to *CBF3*, where it methylates the gene, thereby making the chromatin inaccessible for transcription. (**D**) Timeline of the regulators of the cold response in Arabidopsis at 4 °C. **i**: *SVK*-S reaches a stable peak 8–12 h after initial cold exposure. **ii**: *CBF1* expression peaks 4 h after initial cold exposure (according to some studies). **iii**: *CBF3* expression peaks 3 h after initial cold exposure, then decreases. **iv**: Expression of *ICE*, a *CBF1* activator, reaches a steady peak 1–3 h after initial cold exposure. **v**: Expression of *CBF2*, a *CBF1* repressor, peaks three hours after initial cold exposure, then decreases to almost undetectable levels after 6 h. Created with BioRender.com.

At normal growth temperatures (22 °C), the *SVK*-L isoform makes up the majority of *SVALKA* transcripts. After *SVK*-L transcription, the nascent RNA forms a double-stranded RNA complex with *CBF1* mRNA. *SVK*-L forms a mRNA-*cis*-NAT dsRNA template, which is recognized by DICER-LIKE (DCL) proteins as a substrate (Figure 2A). Recognition of this dsRNA substrate results in the generation of short dsRNA fragments via cleavage. These fragments are then stabilized via methylation from HUA ENHANCER 1 (HEN1). Then, one of the dsRNA fragment guide strands is loaded onto ARGONAUTE1 (AGO1) [21], forming the RNA-Induced Silencing Complex (RISC). Transcript abundance assays suggest that the cleavage products generated by DCL are not amplified, supporting the conclusion that *SVK*-L does not completely silence *CBF1* expression but rather functions to calibrate *CBF1* expression. *CBF1* and *SVK*-L are transcribed simultaneously. Thus, the half-life of the *CBF1* sense RNA is decreased but its transcription levels remain unchanged.

*SVK*-S is the dominant isoform at 4 °C. Maximal *SVK*-S expression begins at 4 h and reaches a steady peak 8–12 h after initial cold exposure. Transcription that is antisense to proximal poly(A) site results in sense/antisense RNAPII competition and collision on both strands. Incoming antisense RNAPII collides with sense RNAPII, resulting in premature *CBF1* transcription termination (Figure 2B). Following the collision event, both the premature *CBF1* mRNA and *SKV*-S transcripts are degraded via a HEN2/exosome mediated mechanism [18].

*SVK*-S has a negative effect on the expression of *CBF1* in response to cold temperatures, which was determined by examining *CBF1* expression changes in Arabidopsis T-DNA insertion lines *svk*-*1*, *uns-1* (uncoupling *SVALKA* 1), and *svk OE* (overexpression). In *svk*-1, *SVK*-S expression is disrupted, and there is a corresponding increase in *CBF1* expression following cold exposure. The same increase in *CBF1* expression occurred in the *uns-1* mutants. In *uns-1* mutants, the insertion increases the distance of *SVALKA* transcription from *CBF1*. Finally, *SVK OE* mutants show decreased *CBF1* levels [17].

*SVK*-S has also been shown to play a role in regulating *CBF3* during the cold response at longer times post-exposure to cold temperature. After *SVALKA* expression peaks 8–12 h after initial exposure to cold stimuli, the Polycomb Repressive Complex 2 (PRC2) is recruited by *SVK*-S to the coding region of the *CBF3* gene. *SVK*-S binds to the CURLY LEAF (CLF) methyltransferase subunit of PRC2 [22]. PRC2 promotes the deposition of the repressive histone mark H3K27me3, silencing *CBF3* gene expression [19] (Figure 2C). This results in *CBF3* transcript levels within the cell decreasing to low levels after approximately 24 h at low temperature conditions. Both *CBF1* and *CBF3* exhibit rapid upregulation in response to cold stress, followed by induction of the *CBF* regulon, which boosts freezing tolerance in Arabidopsis [23,24]. Thus, *SVALKA* negatively regulates both *CBF1* and *CBF3* by inducing the epigenetic silencing of *CBF3* and regulating *CBF1* transcript levels after initial induction.

The timing of lncRNA expression is controlled and employs different mechanisms of gene regulation in response to external environmental cues such as temperature [6,14]. *SVALKA*, for example, employs a DICER based mechanism at normal growth temperatures [18]; at cold temperatures, *SVALKA* employs a PRC2 mediated DNA methylation silencing mechanism after 8 h of initial cold exposure [17] and a transcriptional collision mechanism 8–12 h after exposure [19]. Figure 2D shows a timeline of these mechanisms in the context of the overall plant response to cold stimuli.

*SVALKA* is one of several lncRNAs that participate in the regulation of cold response genes using PRC2 mechanisms. *COOLAIR* [25,26,27], *COLDAIR* [28,29], and *COLDWRAP* [30] are lncRNAs known to regulate flowering locus C (FLC) gene expression in response to cold temperatures. *COLDAIR* has a transient interaction with Curly Leaf (CLF) component of PRC2 [29]. *COLDWRAP* has a long, stable association with PRC2 and continues to be transcribed during and after the full period of cold exposure [30]. *COLDWRAP* and *COLDAIR* coordinate and together form a chromatin loop in the process of silencing through H3K27me3. *COOLAIR* interacts directly with FLOWERING LOCUS A (FCA), which then interacts with PRC2 [31]. FCA has two WW protein interaction domains and two RNA recognition motifs (RRMs) that preferentially bind GU-rich RNA sequences. Thus, through multiple different interactions with PRC2, the time-dependent expression of these lncRNAs regulates the transcriptional response to external cold temperature.

## 3. Dynamic RNA Conformations Mediate lncRNA Function

*COOLAIR* adopts multiple dynamic RNA conformations to regulate FLC [27]. *SVALKA* and the other lncRNAs that participate in regulating the response to cold temperatures may similarly adopt dynamic RNA conformations. *COOLAIR* has been studied by single-molecule chemical probing experiments in vivo [27]. Pac Bio single-molecule sequencing techniques revealed at least three different patterns of nucleotides that were accessible to solvent and chemical reagents that modify nucleotides, such as SHAPE reagents, in the main polyadenylated isoform of *COOLAIR*. The secondary structures were generated using DaVinci, a computational method that emphasizes the results from SHAPE mutational profiles rather than thermodynamic parameters. Three secondary structure models describe conformational ensembles for the main *COOLAIR* isoform. Interestingly, the abundance of each structural model in the ensemble is different at 22 °C and 4 °C. In the shift to lower temperatures, an alternatively spliced isoform of *COOLAIR* predominates and demonstrates evidence of conformational dynamics and structural heterogeneity. The multiple dynamic conformations of *COOLAIR* facilitate its interactions with different protein partners at different stages in its mechanisms of gene regulation.

*SVALKA* may adopt multiple dynamic conformations similarly to *COOLAIR. SVALKA* participates in three types of regulatory mechanisms and may employ different RNA conformations in each mechanism. The dsRNA- and DICER-mediated mechanism and the transcriptional collision mechanism do not necessarily require any higher order RNA structure. However, the switch between the two mechanisms with a change in response to temperature indicates the involvement of other cellular factors and a possible conformational change in *SVALKA*. RNA secondary structure elements in *SVALKA* could facilitate interactions with proteins that mediate this transition in response to low temperature. The most thermodynamically stable conformation for an RNA sequence occurs when it is fully base-paired to its complementary sequence. However, the DICER mediated mechanism in which *SVALKA* forms dsRNA does not occur at 4 °C but rather at 22 °C. A secondary structure that binds protein partners could form a complex that is more thermodynamically stable than the fully base-paired dsRNA conformation. We hypothesize that *SVALKA* binds protein, nucleic acid, or small-molecule partners at lower temperatures and thus increases stability, and that this stable conformation plays a role in the regulation of the cold response of Arabidopsis.

Another *SVALKA* regulatory mechanism involves binding to PRC2. The secondary structure of *COLDWRAP* is important for its interactions with CLF [30]. *XIST* and *HOTAIR* lncRNA also adopt dynamic RNA structures to bind and inhibit EZH2, the mammalian homolog to CLF methyltransferase [30,32,33]. G quadruplex structures in RNA bind PRC2 and regulate its activity [34,35,36]. There are multiple different ways for lncRNA to interact with PRC2 and CLF, each of which may employ different RNA structural motifs. Thus, *SVALKA* may similarly adopt dynamic structural conformations that mediate its interactions with CLF and PRC2.

## 4. Potential for *SVALKA* Gene Regulation in Agricultural Engineering

**Figure 3:**
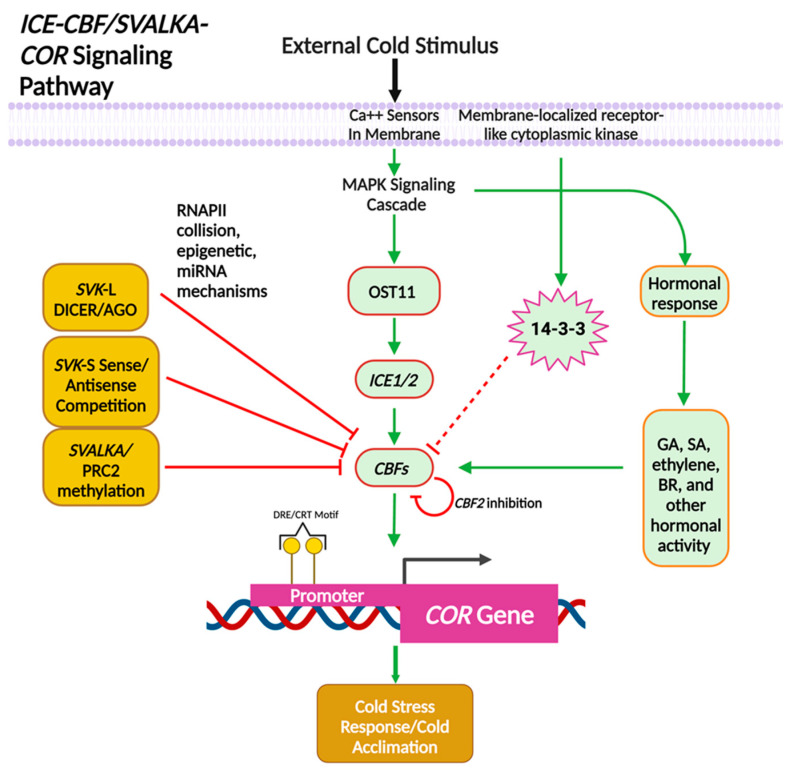
Overview of the *ICE/CBF-SVALKA COR* signaling pathway. Acronyms are as follows: MAPK, mitogen-activated protein kinase; OST1, Open stomata 1; *ICE1/2* Inducer of *CBF* Expression; *CBF*, C-repeat Binding Factor; *COR*, Col regulated genes; CRT/DRE, C-repeat/Dehydration Responsive Element; GA, gibberellin; SA, salicylic acid; BR, brassinosteroids; DICER/AGO, Dicer enzyme ARGONAUTE enzyme; PRC2 Polycomb Repressor Complex 2. Figure is updated and adapted from reference [47]. Created with BioRender.com.

*SVALKA*, *CBF1*, and *CBF3* are part of a larger network of factors that regulate plant response to environmental stresses. *CBF1* is part of the *ICE/COR* pathway that is highly conserved across many plant species [37,38,39]. Figure 3 shows the *ICE-CBF-COR* pathway and the points where *SVALKA* negatively regulates *CBF* expression. CBF2 also negatively regulates *CBF1* and *CBF3* [40,41], as do the *14–3–3* genes whose protein products destabilize CBF proteins after phosphorylation. Cold stress is detected by receptor proteins in the cell membrane that release calcium and trigger a MAPK cascade. The resulting signal transduction activates OST11, which turns on *Inducer of CBF Expression* (*ICE*) genes. ICEs in turn upregulate *CBF* (C-repeat/Dehydration Response) genes, producing CBF protein [42,43,44] and initializing the cold response [38,45,46,47]. Post-transcriptional and/or post-translational modifications (PTMs) increase the binding efficiency and stability of ICE proteins to downstream genes, playing an important role in regulating the *ICE-CBF* signaling pathway during stress response [47,48,49,50]. Further post-translational modification of ICE-CBF proteins, in the form of ubiquitination, improves protein turnover and cold stress tolerance [51]. *ICE-CBF* proteins are regulated hormonally too. They are regulated by the hormonal responses of brassinosteroids (BR), ethylene, gibberellin, and salicylic acid (SA) [52,53]. These hormones regulate basal cold tolerance by controlling the level of *CBF* transcripts. GA in particular also regulates the level of *CBF* transcripts by stimulating the degradation of the DELLA family of local nuclear growth repressive proteins [54,55,56]. The biochemical pathways responding to cold stress utilize protein, plant hormones, and RNA regulatory elements, such as *SVALKA*.

ICE is a protein in the Basic Helix–Loop–Helix (bHLH) family of transcription factors. They contain conserved bHLH domains at their C-terminus [47]. The bHLHs are responsible for regulating the expression of *COR* genes, many of which are a part of the *CBF/COR* regulon. The ICE bHLH domain binds to the *CBF3* promoter, leading to induction of the *CBF* regulon [57,58]. Many different *ICE*-like genes across plant species [59,60] have been investigated in transgenic Arabidopsis to determine their ability to facilitate tolerance to cold stress. ICE homologs have been demonstrated to have conserved motifs and domains that bind to CRT/DRE motifs of CBFs which leads to the induction of downstream *COR* genes [61,62,63,64]. Therefore, the ICE and ICE-like proteins are a crucial first step in the *CBF-COR* regulon and thereby in establishing cold tolerance across multiple species of plants. Upon cold exposure, *ICE* is induced, with significant expression 1–3 h after the initial cold stimulus [65]. In contrast, *SVALKA* expression begins around 4 h after initial cold exposure and reaches a stable peak 8–12 h later [17]. Thus, *CBF* overexpression is both turned on and turned off at the appropriate time through regulation by both protein and RNA.

**Figure 4:**
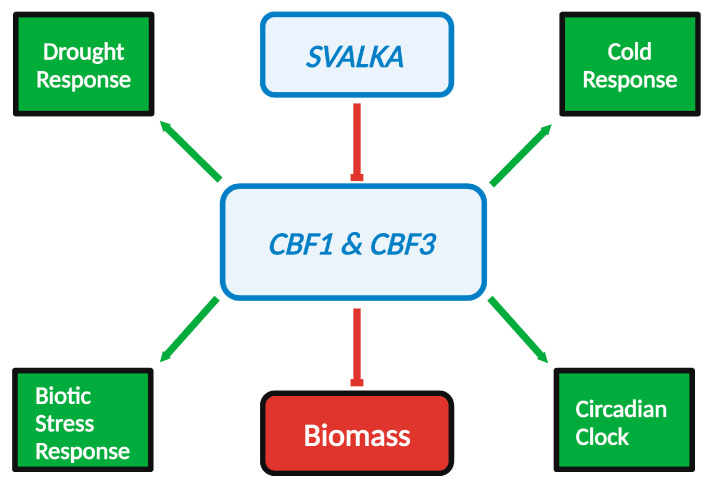
CBF is a master regulator of stress response. As shown by the blunt red arrows, *SVALKA* negatively regulates *CBF1* and *CBF3*, and CBF1 in turn negatively regulates biomass production. As shown by green arrows, CBF1 expression positively regulates genes in the cold response, drought response, biotic stress response, and circadian clock pathways. Created with BioRender.com.

*CBF* expression activates the *COR* regulon as part of many stress response pathways, and thus the regulation of its expression through *SVALKA* and CBF2 has far-reaching implications. The CBFs belong to the APETALA2/Ethylene Responsive (AP2/ERF) superfamily of transcription factors [66], which are essential for cold acclimation and response to several other environmental stresses. *CBF* transcription is responsible for 12–20% of freezing-induced transcription changes in Arabidopsis [42]. CBFs also regulate the stress response to other biotic and abiotic stressors [63,67,68,69,70,71,72] (Figure 4). Members of the CBF/DREB1 protein are characterized by the DSAWR and PKK/KPAGARxKFxETRHP sequences, and an LSWY motif [47]. CBF transcription factors recognize and bind to a cis-regulatory element, the CRT/DRE (C-repeat/dehydration response element) present in the promoters of the *Cold-Regulated* (*COR*) family of genes. The DRE is a 9 bp conserved sequence TACCGACAT which contains the 5 bp CRT core sequence-CCGAC. DRE helps modulate gene expression in response to low temperature, dehydration, and viral stress [45,69,73]. The *COR* genes are part of the larger *CBF* regulon, a family of over 100 genes whose expression is regulated by CBF transcription factors [23,74]. The expression of *COR* genes increases freezing tolerance through multiple mechanisms, such as the synthesis of cryoprotective peptides and the accumulation of solutes such as proline and soluble sugars [45,75]. In addition to the *CBF* regulon, *CBF* genes in Arabidopsis are known to increase cold tolerance through accumulating DELLAs, a family of growth-repressing proteins localized to the nucleus. This is accomplished by decreasing the amount of bioactive gibberellin (GA) in Arabidopsis cells. Gibberellin is a phytohormone which plays an important role in many plant developmental processes and which stimulates the degradation of DELLAs [54]. Therefore, the CBFs are hub transcriptions factors that participate in multiple stress responses.

Because CBFs activate numerous genes and respond to many stresses, the strategies for improving plant resilience through bioengineering CBF1 overexpression must consider a complex network of genes. CBFs respond to drought, temperature, light, and pathogens (Figure 4). Successful strategies must therefore consider the appropriate timing and regulation of expression within the gene networks. Fine-tuning the expression of CBF transcription factors is critical because overexpression of CBFs causes fitness penalties such as decreased biomass, fruit, and seed numbers. Under-expression of CBFs results in a diminished cold response and leads to crop loss [70,71,76,77]. As shown in Figure 4, CBF1 negatively regulates biomass production; thus, simple constitutive overexpression of CBF1 results in cold tolerance but also low biomass production [72,78,79,80,81,82,83,84,85]. Therefore, the timing of turning on and *also turning off genes* for plant resilience is an important factor for successful bioengineering strategies. We will describe three examples of conditional expression of CBF1 regulated by gibberellin (GA), abscisic acid (ABA), or dexamethasone (DEX) that found a better balance between enhancing cold tolerance and maintaining biomass production.

A 2002 study attempted to increase cold resistance in tomato via transformation with *AtCBF1* driven by the potent cauliflower mosaic virus 35S promoter (CaMV) [86]. The expression of *A. thaliana CBF* transcription factors under the control of the cauliflower mosaic virus promoter in transgenic plants leads to strong constitutive expression of the *CBF* regulon and therefore increases freezing tolerance [70,76,77]. The transgenic plants showed dramatically improved cold hardiness compared to the wild type. During normal temperature conditions, the transgenic tomato plants saw decreased fruit set and seed numbers per fruit and exhibited a dwarf phenotype. Normal growth was restored upon exogenous treatment with gibberellic acid (GA). These results demonstrated that strong *CBF1* expression increases cold hardiness but results in decreased crop yield at normal temperature. In contrast to strong constitutive *CBF1* expression, nuanced *CBF1* induction can increase cold tolerance and maintain growth at normal conditions.

A 2003 study used the approach of transforming tomato with *AtCBF1* driven by a stress-responsive promoter complex to ameliorate the dwarf phenotype. Transgenic tomato plants expressed *AtCBF1*, which was governed by three copies of the ABA-responsive complex (ABRC1), a promoter unit which is induced upon binding the stress hormone abscisic acid (ABA) [87]. The result was increased cold tolerance when compared to wild type and nearly identical crop yield. At normal temperatures, the transgenic tomato plants had growth restored to wild-type levels. Therefore, the stress-inducible expression of the *AtCBF1* gene can increase cold tolerance while maintaining growth at normal temperatures [88].

In addition to studying the effects of *AtCBF1* overexpression on cold tolerance, there have been attempts to ameliorate drought stress and postharvest chilling disorder (PCI) via *AtCBF* overexpression. PCI is a physiological condition which leads to global vegetable and fruit crop loss via *AtCBF1* overexpression. In a 2023 study, researchers created transgenic tomato plants with *AtCBF1* under a dexamethasone (DEX)-inducible promoter [89]. Treatment with DEX resulted in a 5- to 11-fold upregulation of *AtCBF1* after 12 h, depending on the concentration of DEX. This DEX chemical inducible system allows for the induction of high levels of *AtCBF1* mRNA in a highly tissue-specific manner. In this study, postharvest chilling disorder was somewhat eased in response to *AtCBF1* expression. Full crop color and volume, however, were not rescued.

The discovery of *CBF1* and its mechanisms of regulation through proteins and plant hormones preceded the discovery of *SVALKA* and mechanisms of negative regulation of *CBF1* and *CBF3* through lncRNA. To our knowledge, no bioengineering strategies have yet used *SVALKA* or any lncRNA to regulate the timing of *CBF* expression. The use of lncRNA rather than externally applied plant hormones has potential advantages. For example, lncRNA can be encoded within the inserted expression vector so that the design includes both the gene for overexpression and its regulatory lncRNA. lncRNA regulation mechanisms do not require additional chemical application and may also avoid potential negative indirect consequences of externally manipulating plant hormone levels. Thus, *SVALKA* and other lncRNAs have the potential for further improving bioengineering designs for improving crop environmental stress resilience.

## 5. Conclusions and Future Possibilities

Cold stress is responsible for over USD 2 billion worth of crop loss globally [90]. Therefore, understanding the natural mechanisms of cold stress response and employing this knowledge to bioengineer crops is important for agriculture improvements during climate change. *SVALKA* negatively regulates *CBF1* and *CBF3* genes, which are central transcription factors for cold response. Because CBF1 also regulates growth and biomass production, turning off *CBF1* expression at the right time is just as important as overexpressing *CBF1*. *SVALKA* utilizes three mechanisms of negatively regulating *CBF1*: a dicer-based mechanism, a collisional transcription mechanism, and a PRC2 epigenetic mechanism. These mechanisms have not yet been adopted into strategies for bioengineering cold tolerance. The use of lncRNA and *SVALKA* in bioengineering has the potential to fine-tune the timing of gene expression to maximize biomass production, cold acclimation, and adaptation to other environmental stresses. Thus, adding lncRNA to the bioengineering toolkit may advance agriculture and RNA biology in the future.
